# Development and Psychometric Evaluation of the Functional Vision Scale for Adults

**DOI:** 10.3390/healthcare14070852

**Published:** 2026-03-27

**Authors:** Keziban Temuçin, Esra Akı

**Affiliations:** Department of Occupational Therapy, Faculty of Health Sciences, Hacettepe University, Ankara 06100, Turkey; esraaki@hotmail.com

**Keywords:** functional vision, scale development, evaluation, validity and reliability

## Abstract

**Objective:** This study aimed to develop and psychometrically evaluate the Functional Vision Scale for Adults (FVSA), an occupation-centred patient-reported outcome measure on daily functioning across key occupational performance domains. **Methods:** The scale development process followed incorporating a comprehensive literature review and expert consultations (n = 10) to establish content validity. An initial item pool of 51 items was refined through expert review and pilot testing (n = 55), resulting in a 33-item scale. The final version was administered to 526 adults (372 without visual problems, 154 with visual problems). Construct validity was evaluated using exploratory and confirmatory factor analysis. Known-group validity was assessed by comparing scores between participants with and without visual problems. Reliability was tested through internal consistency (Cronbach’s α, McDonald’s ω) and test–retest reliability (ICC) in a subsample (n = 62). **Results:** Exploratory and confirmatory factor analyses supported a four-factor structure corresponding to education, activities of daily living (ADLs), social participation and instrumental ADLs (IADLs). Known-group validity was confirmed with statistically significant differences across all subscales (*p* < 0.05). The FVSA Total Score demonstrated excellent reliability, as evidenced by a strong ICC value (ICC = 0.835, *p* < 0.001) supported by high internal consistency coefficients (ω = 0.946; α = 0.933). **Conclusions:** The FVSA is a reliable and valid instrument that provides a comprehensive, occupation-centred assessment of functional vision in adults.

## 1. Introduction

Visual function is an essential component of human performance, deeply influencing individuals’ ability to carry out daily activities, maintain independence, and engage in meaningful social, educational, and occupational roles [1,2]. According to the World Health Organization [3], over 2.2 billion people globally are living with vision impairment or blindness, and this figure is projected to rise with aging populations and the increasing prevalence of chronic diseases such as diabetes. Importantly, while clinical assessments such as visual acuity, contrast sensitivity, or visual field testing provide valuable diagnostic information, they fail to capture the real-world impact of visual impairment on daily life. The concept of functional vision, defined as the effective use of visual abilities to perform everyday tasks, has therefore emerged as a critical domain of assessment in rehabilitation and healthcare settings [4,5,6].

To address the need for patient-centered measurement, several standardized instruments have been developed internationally to assess the functional consequences of visual impairment. Among the most widely used is the National Eye Institute Visual Function Questionnaire (NEI VFQ-25) [7], which evaluates vision-related quality of life across multiple domains, including general vision, near and distance activities, social functioning, mental health, and dependency. The Visual Function Index (VF-14), initially designed to evaluate visual disability in cataract patients, focuses on common daily activities such as reading, watching television, driving, and recognizing people [8]. The Activities of Daily Vision Scale (ADVS) assesses the impact of visual impairment on daily activities, particularly focusing on driving, reading, and general tasks requiring good vision [9]. The Impact of Vision Impairment (IVI) Profile was developed to assess the effects of visual impairment on emotional well-being, mobility, and participation in daily activities [10]. In addition to these general measures, more specific tools have been developed, such as the Low Vision Quality of Life Questionnaire (LVQOL) [11] and Indian Vision Functioning Questionnaire (IND-VFQ-33) [12], each offering valuable insights into the lived experience of vision loss. However, the majority of these tools are anchored in either a medical model or a quality-of-life framework, and they often do not systematically capture the occupationally embedded nature of functional vision as emphasized in rehabilitation disciplines such as occupational therapy.

The American Occupational Therapy Association (AOTA), in its Occupational Therapy Practice Framework: Domain and Process, Fourth Edition, articulates nine key domains of occupational performance: activities of daily living (ADLs), instrumental activities of daily living (IADLs), rest and sleep, education, work, play, leisure, social participation, and health management [13]. Each of these domains relies heavily on functional vision. For example, ADLs such as bathing, dressing, and grooming require visual abilities for object localization and manipulation. IADLs, including meal preparation, medication management, and driving, depend on more complex visual–perceptual and executive functions. Visual function is also indispensable in education, work, play, and leisure, where tasks frequently involve reading, writing, and interacting with digital technologies. Furthermore, social participation and health management require the integration of visual cues for effective communication, self-care, and medical adherence [14,15,16].

Recent systematic reviews have highlighted the need for psychometrically robust, culturally sensitive patient-reported outcome measures (PROMs) that reflect the multidimensional nature of visual impairment and its consequences on functioning [17,18]. However, no available instrument has been explicitly designed to assess functional vision across occupational domains as defined in the AOTA framework. Most existing scales either focus narrowly on specific disease populations, emphasize vision-related quality of life, or fail to integrate the full breadth of occupational performance challenges associated with vision loss. This study aimed to develop and psychometrically evaluate the Functional Vision Scale for Adults (FVSA), an occupation-centred patient-reported outcome measure, and to compare adults with visual problems on daily functioning across key occupational performance domains.

## 2. Method

The study was conducted at the Ophthalmology Clinic of Bursa High Specialization Training and Research Hospital. The study received approval from the Clinical Research Ethics Committee. Ethical protocol number: 2011-KAEK-25 2022/12-15. It was conducted in accordance with the Helsinki Declaration. Written informed consent was obtained from all participants before data collection. The study was conducted and reported in accordance with the COSMIN (COnsensus-based Standards for the selection of health Measurement INstruments) reporting guidelines for studies on measurement properties.

### 2.1. Scale Development Process

The development of the Functional Vision Scale for Adults (FVSA) followed a systematic, multi-stage process aligned with established guidelines for scale construction. A comprehensive literature review was first conducted to define the conceptual framework of functional vision, identify vision-dependent activities, and examine existing adult functional-vision assessment tools. Guided by the Occupational Therapy Practice Framework—Fourth Edition (AOTA, 2020) [19], the research team generated an initial item pool consisting of 51 items that reflected occupation-based and functional-vision–related activities [19].

### 2.2. Content Validity

Content validity was evaluated through two sequential expert-review rounds using the Lawshe technique [20]. A panel of 10 experts from ophthalmology (n = 4), occupational therapy (n = 4), optometry, and rehabilitation sciences (n = 2) independently reviewed the draft scale. Each item was classified as “essential,” “useful but not essential,” or “not necessary.” The Content Validity Ratio (CVR) for each item was calculated using Lawshe’s formula and compared with the critical CVR threshold for 10 experts (CVR ≥ 0.62). Items meeting this value were retained. After incorporating expert feedback across two rounds, the scale was refined to a final set of 33 items.

### 2.3. Pilot Testing

The 33-item version underwent pilot testing with 55 adults aged 18–65 years to evaluate clarity, comprehensibility, and cultural appropriateness. The pilot sample size exceeded commonly recommended methodological minimums for initial testing and was therefore considered sufficient [21]. Based on participant feedback, minor linguistic and structural adjustments were made before proceeding to the main validation study.

### 2.4. Participants

A total of 526 participants were included in the psychometric evaluation and categorized into two main groups. The Test Group consisted of 372 adults without any ophthalmologist-diagnosed visual problems. The Known Group included 154 adults diagnosed with visual problems known to affect functional vision, such as cataract, glaucoma, or age-related macular degeneration. Inclusion criteria required participants to be between 18 and 65 years of age, able to understand Turkish, and willing to provide voluntary informed consent. For the Known Group, an ophthalmologist-confirmed visual problems were required. Exclusion criteria encompassed permanent physical limitations affecting activity participation, cognitive impairment as indicated by a Mini-Mental State Examination (MMSE ≤ 24), insufficient Turkish proficiency, or refusal to participate.

### 2.5. Data Collection

Our study was conducted in person with patients diagnosed with any visual impairment affecting functional vision and healthy participants who presented to the Outpatient Clinic of Bursa Specialized Training and Research Hospital for routine examinations. Due to time constraints, contact information was collected from only a small number of healthy participants. Scale questions were asked verbally over the phone, and the responses were recorded. To optimize the efficiency and quality of interaction in online meetings, voice calls were preferred for this process. There were no issues with the intelligibility of the questions. Data collection began in June 2024 and was completed in January 2025. To assess test–retest reliability, a subsample of 62 participants from the Test Group completed the FVSA twice, with a two-week interval between assessments. All participants also completed a sociodemographic form along with the FVSA.

### 2.6. Psychometric Evaluation

Construct validity was examined using both Exploratory Factor Analysis (EFA) to identify the underlying factor structure and Confirmatory Factor Analysis (CFA) to evaluate the model fit of the derived structure.

Known-group validity was assessed by comparing FVSA scores between adults with ophthalmologist-diagnosed visual problems and those without, thereby evaluating the scale’s discriminatory ability based on expected group differences.

Reliability was evaluated through internal consistency coefficients, including Cronbach’s alpha and McDonald’s Omega, and through test–retest reliability using intraclass correlation coefficients (ICC) computed from the two assessment time points.

### 2.7. Statistical Analyses

All the analyses were conducted using SPSS software (version 24.0; IBM Corp., Armonk, NY, USA) and Jamovi (2.7.16 version), which operates on the R statistical environment [22]. A significance level of *p* < 0.05 was adopted for all analyses. The Shapiro–Wilk test was used to evaluate the normality of numerical variables. Descriptive statistics were presented as means and standard deviations (M ± SD) for normally distributed continuous variables, and as frequencies and percentages for categorical variables. Item analysis was conducted to evaluate item–total correlations and item discrimination coefficients, and to determine the contribution of each item to the overall scale structure. Construct validity of the scale was evaluated using a cross-validation strategy involving both Exploratory Factor Analysis (EFA) and Confirmatory Factor Analysis (CFA). To ensure the independence of the findings and to enhance the generalizability of the model, the total sample (n = 526) was randomly divided into two independent subsamples (n = 263 for each). The first subsample (n = 263) was used for the exploratory phase. Prior to factor extraction, the suitability of the data was assessed using the Kaiser–Meyer–Olkin (KMO) measure of sampling adequacy and Bartlett’s Test of Sphericity. Factor extraction was performed using Principal Axis Factoring, as this method focuses on shared variance and is more appropriate for identifying underlying latent constructs compared to component-based approaches such as Principal Component Analysis. Given that the theoretical domains of the scale were expected to be conceptually related, an oblique rotation method was applied to allow for correlations among factors. The number of factors to retain was determined based on eigenvalues greater than 1, examination of the scree plot, and the conceptual interpretability of the factor structure. Items with factor loadings below 0.40 or those demonstrating substantial cross-loadings were carefully reviewed and considered for removal based on both statistical criteria and clinical judgment. The second independent subsample (n = 263) was used to perform CFA in order to verify the factor structure identified in the exploratory phase. Model fit was evaluated using multiple goodness-of-fit indices, including the chi-square (χ^2^) test, Comparative Fit Index (CFI), Tucker–Lewis Index (TLI), and Root Mean Square Error of Approximation (RMSEA). Known-group validity was examined by comparing FVSA scores between individuals with ophthalmologist-confirmed visual problems and those without. Depending on the normality of data distributions, group comparisons were performed using the independent samples *t*-test to determine the ability of the scale to discriminate between groups expected to differ in functional vision. Internal consistency reliability was assessed using Cronbach’s alpha and McDonald’s Omega coefficients. Test–retest reliability was evaluated using the Intraclass Correlation Coefficient (ICC), calculated from repeated measurements obtained from a subsample of participants after a two-week interval.

## 3. Results

The mean age of participants in the visual problem known group was 37.74 years (SD = 11.48), while the test group had a mean age of 36.36 years (SD = 10.67) (See Table 1).

The content validity process was conducted in two iterative rounds of expert review. In the first round, items with a Content Validity Ratio (CVR) below the threshold value of 0.62 were identified and removed. Several items, including “I can read the prescription label on a medication bottle” (CVR = −0.60), “I can read newspaper and book text beyond headlines” (CVR = 0), and “I can read street names and store signs when I am outside” (CVR = 0), did not meet the required level of expert agreement and were therefore excluded. In addition to CVR-based removal, items with overlapping or redundant content were reviewed and either merged or reworded. Furthermore, new items were generated based on expert feedback to improve content coverage and conceptual completeness. Following these revisions, the item pool was refined to 45 items and submitted for a second round of expert evaluation. During the second round, 12 additional items fell below the CVR threshold (CVR < 0.62) and were subsequently removed. Examples of these items include “I can see signs, labels, and numbers when using public transportation” (CVR = 0.40), “I can read price labels on products in stores” (CVR = 0.60), and “I can read a wristwatch to tell the time” (CVR = 0.60). After this stage, further minor revisions and item combinations were carried out to reduce redundancy and enhance conceptual clarity. As a result of this systematic refinement process, the final item pool consisted of 33 items, all of which demonstrated acceptable content validity (see Supplementary Materials S1).

### 3.1. Item Analysis

Item analysis indicated that corrected item–total correlations ranged from 0.347 (I6) to 0.721 (I9). All values exceeded the commonly accepted threshold of 0.30, suggesting that each item contributed meaningfully to the overall construct and demonstrated adequate internal consistency.

### 3.2. Validity

Exploratory Factor Analysis: The suitability of the data for factor extraction was confirmed by a Kaiser–Meyer–Olkin (KMO) value of 0.869, indicating meritorious sampling adequacy. Bartlett’s Test of Sphericity was also statistically significant (χ^2^ = 5164, df = 528, *p* < 0.001), supporting the appropriateness of the correlation matrix for factor analysis. In addition, the Individual Measure of Sampling Adequacy (MSA) values for all 33 items ranged from 0.706 to 0.936, exceeding the recommended threshold of 0.50. Item I25 demonstrated a meaningful secondary cross-loading on Factor 2 (loading = 0.435). Based on methodological stability and theoretical alignment, I25 was reassigned to Factor 2, resulting in a more coherent and robust four-factor structure for subsequent analyses. These four factors collectively explained 43.5% of the total variance in this independent subsample. Confirmatory factor analysis (CFA) was then conducted to verify the accuracy of this proposed four-factor model (see Table 2).

**Figure 1:**
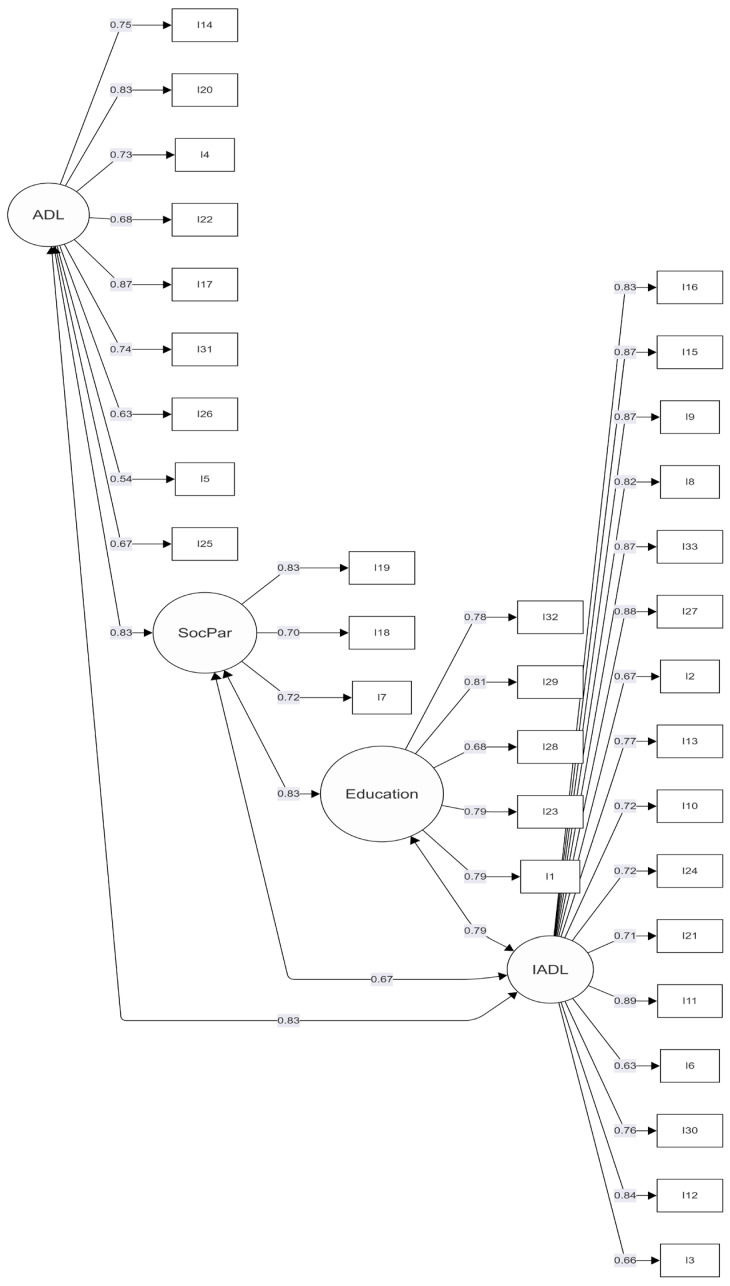
Path diagram about confirmatory factor analysis.

Confirmatory Factor Analysis (CFA): The four-factor structure obtained from the exploratory factor analysis was tested using confirmatory factor analysis. The chi-square to degrees of freedom ratio (CMIN/DF = 2.67) was within the commonly accepted range (2–3), indicating an acceptable level of model fit relative to model complexity. Incremental fit indices also suggested an adequate fit, with the Comparative Fit Index (CFI = 0.960), Normed Fit Index (NFI = 0.947), and Tucker–Lewis Index (TLI = 0.963) meeting recommended thresholds. Furthermore, the Root Mean Square Error of Approximation (RMSEA = 0.068) was within the acceptable range (0.05–0.08), indicating a reasonable approximation error and overall model adequacy [23,24]. Following the confirmatory factor analysis, the factor structure was reviewed, and the factors were subsequently renamed and renumbered to better reflect their conceptual meanings. As a result of this process, Factor 1 was defined as Education, representing items related to learning and academic engagement. Factor 2 was labeled Activities of Daily Living (ADL), encompassing core self-care and routine daily activity items. Factor 3 was named Social Participation, capturing items associated with interpersonal interaction, social engagement, and community involvement. Finally, Factor 4 was identified as Instrumental Activities of Daily Living (IADL), representing more complex daily tasks requiring higher-level cognitive and functional abilities (See Table 3 and Figure 1).

Known-Group Validity: Independent samples *t*-test results demonstrated significant differences between the test group (0) and the known group (1) across all domains (*p* < 0.05). Consistent with the scoring criteria, in which higher scores indicate greater functional difficulty, participants in the known group had significantly higher Education scores (Mean = 8.05 vs. 6.97; *p* < 0.001), ADLs scores (Mean = 5.64 vs. 5.37; *p* = 0.009), and Social Participation scores (Mean = 3.76 vs. 3.47; *p* = 0.020) compared with the test group. Similarly, IADL scores (Mean = 23.00 vs. 20.57; *p* < 0.001) and the FVSA Total Score (Mean = 40.45 vs. 36.39; *p* < 0.001) were significantly higher in the known group. These findings indicate that individuals with ophthalmologist-confirmed visual problems reported greater functional-vision difficulties across all domains of the FVSA, thereby confirming the scale’s known-group validity (See Table 4).

Internal consistency and test–retest reliability analyses demonstrated that the FVSA and its subscales had acceptable to excellent reliability (Table 5). The Education subscale showed acceptable temporal stability (ICC = 0.625, *p* < 0.001) supported by adequate internal consistency (ω = 0.746; α = 0.727). The ADLs subscale demonstrated acceptable test–retest reliability (ICC = 0.649, *p* < 0.001) and good internal consistency (ω = 0.813; α = 0.797). The Social Participation subscale yielded the lowest ICC value among the subscales (ICC = 0.503, *p* = 0.001); however, its internal consistency coefficients (ω = 0.703; α = 0.673) indicated an acceptable level of reliability. The IADLs subscale demonstrated good test–retest reliability (ICC = 0.756, *p* < 0.001) along with strong internal consistency (ω = 0.921; α = 0.905). Finally, the FVSA Total Score exhibited excellent reliability, with a high ICC value (ICC = 0.835, *p* < 0.001) and very strong internal consistency (ω = 0.946; α = 0.933). (See Table 5).

## 4. Discussion

The findings of the current study demonstrate that the FVSA possesses strong psychometric properties, including robust content validity, construct validity, known-group discrimination, internal consistency and test–retest reliability.

The scale encompasses four core domains identified in the AOTA framework, with each corresponding subscale addressing specific areas of occupational performance. Education includes items related to academic and learning activities; Activities of Daily Living covers fundamental self-care tasks such as grooming and dressing; Social Participation includes items evaluating interpersonal communication and community involvement; and Instrumental Activities of Daily Living focuses on more complex daily tasks such as cooking, shopping, and managing household responsibilities. When compared with the subdimensions of existing instruments, the FVSA’s four-factor structure demonstrates a level of comprehensiveness that is comparable to, and in some domains surpasses, other widely used functional vision measures. The NEI VFQ-25, for instance, is designed to assess the impact of various eye diseases on vision-related quality of life. The 25-item version typically consists of 12 subscales: general health, general vision, ocular pain, near activities, distance activities, social functioning, mental health, role difficulties, dependency, driving, color vision, and peripheral vision [7]. Similarly, the Impact of Vision Impairment (IVI) questionnaire includes three major dimensions: emotional well-being, mobility and independence, and reading and accessing information [10]. The Low Vision Quality of Life Questionnaire (LVQOL), developed specifically for individuals with low vision, includes 25 items grouped into four categories: distance vision and mobility, adjustment, reading and fine work, and activities of daily living [11]. While these instruments provide valuable insights, their subscales do not cover functional domains such as basic and instrumental activities of daily living (e.g., personal care, household management, transportation) as explicitly and systematically as the FVSA. For instance, instruments like the LVQOL combine these tasks into a single, broad ADL category, which limits the ability to distinguish between fundamental self-care activities (ADLs) and more complex community and home management tasks (Instrumental ADLs). Additionally, many of these instruments were developed primarily for older adults and therefore may not fully capture the functional demands of younger or working-age populations.

The item generation and refinement process of the FVSA incorporated both expert consensus and patient voice, aligning with best practices in PROM development and increasing the content validity of the scale [18,20]. The FVSA ensures high ecological validity and contextual relevance increasingly emphasized in patient-centered care [14,15]. However, although the FVSA was inspired by the AOTA framework, it does not encompass all nine domains of occupational performance outlined in the framework. For instance, some items related to leisure (e.g., “I have trouble distinguishing details while engaging in my hobbies (e.g., crafts, gardening, painting)”) and productivity (e.g., “I do not experience vision-related problems in my work life”) were removed following expert consensus. These exclusions may reflect the prioritization of domains most functionally impacted by visual problems and those considered most relevant to the target population.

Confirmatory factor analysis supported a four-factor model with acceptable to good fit indices (CFI = 0.960, NFI = 0.947, TLI = 0.963, RMSEA = 0.068), consistent with commonly recommended threshold values aligning with Hu and Bentler’s (1999) [23] recommended thresholds. Notably, while previous instruments such as the IVI and the LVQOL provide domain-specific insight, they often center on emotional or social aspects of quality of life without explicitly structuring items according to occupational domains. In contrast, the FVSA anchors each item in functionally meaningful occupations, consistent with the core principles of occupational therapy and recent recommendations advocating for domain-specific construct clarity in PROM development [17]. Moreover, known-group validity results confirmed the ability of the FVSA to discriminate between adults with and without visual problems (*p* < 0.05 for all subscales), providing empirical support for the scale’s clinical sensitivity.

The internal consistency of the FVSA was strong, with a Cronbach’s alpha of 0.933 for the total scale and acceptable to excellent alpha coefficients across the subscales. McDonald’s omega coefficients further supported this pattern of reliability, ranging from 0.703 to 0.946, indicating that the FVSA demonstrates a stable and coherent latent structure across domains. These findings are comparable to those reported in the validation of other widely used instruments such as the NEI VFQ-25 (α = 0.88) and the VF-14 (α = 0.85) [8]. Test–retest reliability analyses demonstrated acceptable to excellent temporal stability across the subscales, with ICC values ranging from 0.503 to 0.835. These results are comparable to the test–retest performance of instruments such as the IND-VFQ-33 (alpha > 0.80; item-total correlations 0.54–0.86) [12] and indicate that the FVSA also provides consistent measurements over time.

Despite these strengths, the group of participants with visual problems was heterogeneous, which enhances generalizability but may obscure condition-specific functional patterns. The unequal distribution of demographic characteristics within the sample, particularly across gender groups, limited the ability to fully assess measurement invariance. This was therefore considered an important limitation of the present study. Future research should aim to include more balanced and representative samples. This would enable the application of multi-group confirmatory factor analysis (multi-group CFA) across different demographic characteristics, allowing for a more comprehensive and robust examination of measurement invariance.

In addition, online interview methods are frequently used in current scientific studies to reach larger sample groups. In our study, we employed both online and in-person interview methods. To enhance the efficiency of the online interviews, they were conducted via voice calls. For future studies, the effects of different interview methods could be discussed, or a single interview method could be selected.

## 5. Conclusions

The Functional Vision Scale for Adults (FVSA) was developed as an occupation-centered, psychometrically sound instrument to assess the real-world impact of visual conditions on daily functioning. Grounded in the AOTA’s occupational performance framework, the FVSA captures a wide range of activity limitations across domains such as Education, Activities of Daily Living, Social Participation and Instrumental Activities of Daily Living (IADLs). The scale demonstrated strong content and construct validity as well as high internal consistency, supporting its robustness as a functional vision assessment tool. The FVSA provides a valuable contribution to both clinical assessment and research, particularly in rehabilitation contexts aiming to promote participation and autonomy in individuals with visual problems. Future studies should examine its applicability across diverse cultural and diagnostic populations.

## Figures and Tables

**Table 1:** Demographic characteristics of participants.

Variable	Category	Known Group (n = 154)	Test Group (n = 372)
Gender	Female	124	283
	Male	30	89
Education Level	Illiterate	1	1
	Primary school	4	13
	Middle school	20	78
	High school	27	130
	University	78	169
	Postgraduate	24	48
Employment Status	Full-time	79	182
	Part-time	8	13
	Retired	6	9
	Unemployed	61	168
Vision-intensive Day	Yes	96	-
	No	58	-
Eyeglasses Use	Yes	91	-
	No	63	-
Light Sensitivity	Yes	86	-
	No	68	-

**Table 2:** Exploratory Factor Analysis Results.

	Components				
	1	2	3	4	5
I1	0.484				
I23	0.719				
I28	0.668				
I29	0.418				
I32	0.662				
I4		0.697			
I5		0.587			
I14		0.429			
I17		0.719			
I20		0.483			
I22		0.444			
I26		0.841			
I31		0.662			
I7			0.653		
I18			0.439		
I19			0.771		
I2				0.496	
I3				0.483	
I6				0.415	
I8				0.667	
I9				0.533	
I10				0.742	
I11				0.808	
I12				0.785	
I13				0.475	
I15				0.853	
I16				0.551	
I21				0.523	
I24				0.558	
I27				0.567	
I30				0.402	
I33				0.673	
I25		0.435			0.510

**Table 3:** Confirmatory Factor Analysis (CFA) results.

Parameters	Abbreviation	Acceptable Range of Fit	Value
Chi-square fit test	CMIN/DF	2≤CMIN/DF≤3	2.669
Comparative Goodness of Fit Index	CFI	0.95≤CFI≤0.97	0.960
Normed Fit Index	NFI	0.90≤NFI≤0.95	0.947
Tucker–Lewis Index	TLI	TLI ≥0.95	0.963
Root Square Mean Error of Approximation	RMSEA	0.05≤RMSEA≤0.08	0.068

**Table 4:** Known group validity results.

Variable	Diagnosis Status	n	Mean	SD	t	df	*p*
Education	0	372	6.97	1.77	−5.266	524	<0.001
	1	154	8.05	2.84			
ADLs	0	372	5.37	0.89	−2.608	524	0.009
	1	154	5.64	1.46			
Social Participation	0	372	3.47	1.16	−2.326	524	0.020
	1	154	3.76	1.54			
IADLs	0	372	20.57	4.04	−4.775	524	<0.001
	1	154	23.00	7.56			
FVS Total	0	372	36.39	6.82	−4.843	524	<0.001
	1	154	40.45	12.25			

0: Test Group; 1: Known Group; *p* < 0.05.

**Table 5:** Findings on internal consistency test–retest reliability. Test–Retest Reliability and Internal Consistency.

	ICC	McDonalds ω	Cronbach α	*p*
Education	0.625	0.746	0.727	<0.001
ADLs	0.649	0.813	0.797	<0.001
Social Participation	0.503	0.703	0.673	0.001
IADLs	0.756	0.921	0.905	<0.001
FVSA Total	0.835	0.946	0.933	<0.001

ICC: Intraclass correlation coefficient; *p* < 0.001; ADL: Activities of Daily Living; IADLs: Instrumental Activities of Daily Living; FVSA: Functional Vision Scale for Adults.

## Data Availability

Data from this study is not available because of ethical reasons.

## Supplementary Materials

The following supporting information can be downloaded at: https://www.mdpi.com/article/10.3390/healthcare14070852/s1, Supplementary Materials S1: Items Removed After the First Expert Review; Items Removed After the Second Expert Review; Item Wording Before and After the Pilot Study.
