# Supplementary material for: Development and Psychometric Evaluation of the Functional Vision Scale for Adults
Source: Healthcare (Basel). 2026 Mar 27;14(7):852. doi: 10.3390/healthcare14070852 (PMC13073657; doi:10.3390/healthcare14070852)
Supplement: Supplementary file 1 [file healthcare-14-00852-s001.zip › healthcare-4202492-supplementary.pdf]

Supplement S1

Items Removed After the First Expert Review

Item Number	Item	Content Validity Ratio (CVR)
2	I have difficulty seeing the images or subtitles clearly when watching television.	-0.60
3	I can read the text in books and newspapers beyond the headlines.	0
4	When I go outside, I can read street names and store signs.	0
14	I can reach for the object I want without making errors in judging distance or depth.	0
18	I can perceive stimuli from different visual fields (right-left, up-down) and proceed without difficulty.	0
22	I have trouble distinguishing details while engaging in my hobbies (e.g., crafts, gardening, painting).	0
23	Even when dazzled by car lights, I do not experience functional problems.	-0.40
30	I can comfortably watch television and see what I'm watching clearly.	0
36	I have difficulty cutting my nails.	0
46	I have difficulty recognizing familiar people across the street.	-0.40

48	My vision abilities do not contribute to situations that make me angry.	-0.20
49	My vision abilities do not contribute to situations in which I fail.	0
50	My vision abilities do not contribute to situations that make me unhappy.	0
51	My vision abilities do not contribute to my need for others' help.	0

Items Removed After the Second Expert Review

Item Number	Item	Content Validity Ratio (CVR)
2	I can see signs, labels, and numbers when traveling by public transportation.	0.40
4	I can read the text on product labels in stores.	0.60
12	I have no vision-related difficulties when going up or down stairs.	0.20
14	I can find my friends' houses or restaurants on my first visit by recognizing the address.	0
18	I can fasten my coat zipper or button using vision.	0.40
23	I have difficulty reading written documents in the work environment (e.g., emails, reports, notes).	0.20
30	I can read the name tag of a staff member in an institution or store.	0.40

35	I struggle to clearly follow written or visual materials presented during meetings.	0.60
40	I can choose my clothes according to season and color.	0
41	I can use a computer without requiring special software (such as JAWS, Dolphin, etc.).	0.20
42	I can see and correctly pierce my food with a fork.	0.40
43	I do not experience vision-related problems in my work life.	0.20

Item Wording Before and After the Pilot Study

Item Number	Before Pilot Study	After Pilot Study
1	I can read the indicators on home appliances (oven, washing machine, etc.).	I can read the indicators on the surface of home appliances (oven, washing machine, etc.).
10	I can perform tasks such as chopping and cutting using my vision.	I can perform tasks such as chopping and cutting by seeing.
12	I can do tasks requiring detailed close-up vision (sewing, screwing, repairs, etc.).	I can do tasks requiring close-up vision (sewing, screwing, repairs, etc.).
21	I can see the keyhole and insert the key.	I can see the keyhole and unlock the door with the key.
24	I can plug an electrical plug into the socket by looking.	I can plug an electrical plug into the socket by seeing.

26	I can apply toothpaste to the toothbrush by looking.	I can apply toothpaste to the toothbrush by seeing.
